# Protecting children from tobacco products in retail environments: A review of Australian tobacco control laws

**DOI:** 10.1111/dar.14033

**Published:** 2025-03-09

**Authors:** Tess Rooney, Michaela Okninski, Kylie Morphett, Bernadette Richards, Coral Gartner

**Affiliations:** ^1^ School of Public Health The University of Queensland Brisbane Australia; ^2^ NHMRC Centre of Research Excellence on Achieving the Tobacco Endgame The University of Queensland Brisbane Australia; ^3^ School of Medicine The University of Queensland Brisbane Australia

**Keywords:** health law, health policy, tobacco retailing

## Abstract

**Issues:**

Tobacco retailing remains highly prevalent in Australia and so represents a potential source of exposure to tobacco marketing for children, despite national laws that restrict tobacco advertising, promotion and sponsorship. This study sought to answer the question of how comprehensively the current Australian regulatory framework protects children from potential exposure to tobacco marketing in retail settings.

**Approach:**

We reviewed and summarised Australian tobacco control laws (federal, state and territory) for provisions related to protecting children from supply or advertising/promotion of tobacco products in retail settings. We analysed the laws for differences between jurisdictions and considered how comprehensively they protect children from exposure to tobacco product marketing in retail environments.

**Key Findings:**

We found several gaps in the laws that leave children exposed to tobacco product marketing in retail environments. For example, some jurisdictions allow children to sell tobacco products and some do not undertake controlled purchase operations to monitor compliance. No jurisdiction currently restricts the location or number of tobacco retailers, or the types of retailers who can sell tobacco (including toy stores).

**Implications:**

There are opportunities to strengthen tobacco retailing regulations in Australia to better distinguish tobacco from everyday consumer products and to protect children from tobacco marketing in retail environments.

**Conclusions:**

Even in countries with strong tobacco advertising and promotion restrictions, such as Australia, weaknesses in tobacco laws leave children exposed to tobacco product retailing in ways that normalises tobacco product sales and use. Tobacco retailing laws should be strengthened to denormalise commercial tobacco products.

**Key Points**

- Jurisdictional differences exist in how tobacco retailing is regulated in Australia.
- There are few restrictions on practices, such as window toy displays in tobacconists or preventing stores that predominately sell children's products from retailing tobacco products, and no proximity limits on the location of tobacco retailers.
- There are many opportunities to strengthen tobacco control laws in Australia to protect children from being exposed to tobacco sales, marketing and advertising in retail environments.

## 1 INTRODUCTION

Preventing the initiation of smoking by young people is a global priority and a common justification for restricting tobacco marketing. The World Health Organization Framework Convention on Tobacco Control (FCTC) specifically cites concern about tobacco product use by children, adolescents and young girls, and references the Convention of the Rights of the Child ‘to the enjoyment of the highest attainable standard of health’ in its preamble, while FCTC Article 16 addresses sales to and by minors [1]. There are prominent examples over many years of references to protection of youth when introducing laws to restrict tobacco marketing. In the United States, President Obama described the *Family Smoking Prevention and Tobacco Control Act* as a ‘law that will reduce the number of American children who pick up a cigarette and become adult smokers’ [2]. Similarly, in Canada, the *Cracking Down on Tobacco Marketing Aimed at Youth Act* sought to specifically prevent marketing strategies that targeted minors [3]. In Australia, the need to protect youth from tobacco marketing was cited in consultation documents associated with the *Public Health (Tobacco and Other Products) Bill* 2023 and the *Public Health (Tobacco and Other Products) Regulations* 2023 [4]. While youth smoking has declined in Australia, it remains a significant concern, with 13.5% of school students aged 12–17 reporting ever smoking in 2022/2023, including 20.4% of those aged 16–17 [5].

Banning tobacco advertising, promotion and sponsorship is an effective measure to decrease smoking uptake among youth [6] and is supported by Article 13 of the FCTC [1]. Some 42 countries have implemented some form of tobacco advertising, promotion and sponsorship ban, generally consisting of bans on mass media advertising and point‐of‐sale marketing and product displays, and indirect advertising, such as sponsorship and promotional discounts [7]. However, tobacco companies continue to aggressively oppose marketing restrictions and innovate their marketing strategies [8], particularly in ‘dark markets’ [9], that is, those that have eliminated most opportunities for overt advertising of tobacco products, such as Australia, to ‘maximise existing loopholes’ to circumvent tobacco advertising, promotion and sponsorship restrictions [8]. Hence, a comprehensive approach to protecting youth from taking up smoking needs to address a wide range of tobacco marketing strategies, including packaging, product features (e.g., flavours), affordability (including small pack sizes to reduce upfront pack price) and the location and activities of tobacco retailers (e.g., product displays and signage) [6, 10, 11, 12, 13].

Bricks‐and‐mortar tobacco retailers remain a key marketing channel where Australian children are potentially exposed to tobacco marketing. As highlighted by Freeman et al., ‘Tobacco retailers are not neutral tobacco purchasing outlets, they are a key pillar in the promotion of tobacco products’ [7]. Widespread exposure to tobacco products sustains the tobacco epidemic by enabling not only easy product access but also increasing environmental cues to purchase and use tobacco [9]. Exposure to tobacco retail outlets can undermine smoking cessation attempts and trigger relapse among adults who have recently quit [9, 14]. Research into the relationship between tobacco retailer density and proximity and smoking behaviour found smoking decreased with reduced exposure to tobacco retail outlets [15]. For children and minors, exposure to tobacco retailing is associated with increased likelihood of experimentation, initiation and uptake of smoking [13, 15]. A meta‐analysis found that minors exposed to point‐of‐sale tobacco promotions were 1.6 times more likely to have tried smoking, than those with no exposure [13]. The role of point‐of‐sale marketing displays in undermining tobacco denormalisation strategies and increasing environmental cues that encourage smoking was a justification for marketing restrictions included in the FCTC [1, 10]. However, even in jurisdictions with bans on point‐of‐sale marketing and tobacco product displays, signage alerting customers to the availability of tobacco products, tobacco product price lists or general awareness that the products are available for sale may still act as a trigger to purchase tobacco and normalise its use. For example, following implementation of a retail display ban in Western Australia (WA), spontaneous purchases of tobacco products from supermarkets were reduced from 28.2% pre‐ban to 19.8% post‐ban [16]. This suggests that even with a retail display ban in place, one‐in‐five people who purchased tobacco from a supermarket had not intended to buy tobacco before entering the store. Research in Sydney, Australia with people who were trying to quit smoking found that viewing images of tobacconists, store tobacco price lists and related signage triggered thoughts of smoking and impulse purchasing [9].

Australia is considered a country with well‐developed tobacco control laws and has been a leader in implementing tobacco advertising, promotion and sponsorship restrictions, with extensive bans implemented in federal legislation [4, 9]. However, as a federated country where tobacco control is a shared responsibility between the Commonwealth Government and the six states (Queensland, New South Wales [NSW], Victoria, Tasmania, South Australia [SA] and WA) and two territories (Australian Capital Territory and Northern Territory [NT]), which are sub‐national regions/provinces, variations in tobacco control laws between jurisdictions exist. Tobacco control at the retailer level is generally the responsibility of the states and territories, each of which has established its own regime regarding the regulation of tobacco retailing. This arrangement adds a layer of complexity to any attempt to comprehensively close regulatory gaps that enable targeting of youth and that undermine tobacco denormalisation.

In this study, we reviewed and summarised Australian tobacco control laws that regulate the marketing of smoked tobacco products in physical retail premises to answer the following research question: How comprehensively does the current regulatory framework protect children from potential exposure to tobacco marketing in retail settings?

## 2 METHODS

We identified the key legislation (the laws made by parliaments), and subordinate legislation (laws made under delegated authority conferred by an Act of Parliament, such as regulations) that regulate the supply of tobacco products federally and in each Australian state and territory. Government health department websites were searched using tobacco retailing terms (i.e., ‘tobacco retailer’ and ‘tobacco retail licence’), and local tobacco control researchers and health departments were contacted for clarification of details as needed. We reviewed the key legislation and other information sources (e.g., guides, factsheets, decisions from administrative tribunals, reports and policy documents published on health department websites) for provisions related to protecting children from tobacco marketing in bricks‐and‐mortar retail stores and coded each jurisdiction in a Microsoft Word table against an initial list of pre‐defined legal provisions based on the author team's knowledge of Australian tobacco control laws and public health measures that are relevant to protecting children from tobacco marketing [10, 11, 12]. As the review progressed the coding was expanded and refined, and additional relevant provisions were identified, into the following categories: (i) retailer licensing; (ii) specialist tobacconist retailers; (iii) sale to minors prohibited; (iv) purchasing on behalf of minors prohibited; (v) sales by minors prohibited; (vi) controlled purchase operations; (vii) minimum pack size; (viii) flavour prohibitions; (ix) packaging restrictions; (x) sale of toys/confectionery resembling tobacco products prohibited; (xi) restrictions on display/sale of toys/confectionery/children's products in close proximity to tobacco or price boards; (xii) display of tobacco and related products prohibited; (xiii) tobacco advertising prohibited; and (xiv) any other restrictions imposed on tobacco retailing related to protection of children. All legislation was checked for the most recent version, and the results were updated accordingly in November 2024 and February 2025.

## 3 RESULTS

Table 1 lists the abbreviations used in this review for each of the key legislations that regulates tobacco marketing, promotion and supply in Australia.

**TABLE 1:** Key abbreviations used in reporting of results.

Abbreviation	Full references
Cth Act	*Public Health (Tobacco and Other Products) Act 2023 (Cth)*. No. 118, 2023; C2024C00259 (C01). Effective: 1 July 2024 [cited 2024 Aug 8]. Available from: https://www.legislation.gov.au/C2023A00118/latest/text
Cth Reg	*Public Health (Tobacco and Other Products) Regulations 2024 (Cth)*; F2024L00415. Effective 28 March 2024 [cited 2024 Aug 16]. Available from: https://www.prod.legislation.gov.au/F2024L00415/latest/text
ACT Act	*Tobacco and Other Smoking Products Act 1927 (ACT)*. A1927‐14 Republication No. 37. Effective: 10 December 2022 [cited 7 Dec 2023]. Available from: www.legislation.act.gov.au/a/1927‐14/
NSW Act	*Public Health (Tobacco) Act 2008 (NSW)*. No. 94. Effective: 30 October 2023 [cited 7 Dec 2023]. Available from: https://legislation.nsw.gov.au/view/whole/html/inforce/current/act‐2008‐094
NSW Reg	*Public Health (Tobacco) Regulation 2022 (NSW)* [2022–469]. Effective: 27 May 2024 [cited 17 Novr 2024]. Available from: https://legislation.nsw.gov.au/view/html/inforce/current/act‐2008‐094
NT Act	*Tobacco Control Act 2002 (NT)*. Reprint: REPT040. Effective: 20 November 2020 [cited 7 Dec 2023]. Available from: www.legislation.nt.gov.au/en/Legislation/TOBACCO‐CONTROL‐ACT‐2002
NT Reg	*Tobacco Control Regulations 2002 (NT)*. Effective: 14 April 2020 [cited 7 Dec 2023]. Available from: www.legislation.nt.gov.au/Legislation/TOBACCO‐CONTROL‐REGULATIONS‐2002
Qld Act	*Tobacco and Other Smoking Products Act 1998 (Qld)*. Effective 1 September 2023 [cited 7 Dec 2023]. Available from: www.legislation.qld.gov.au/view/whole/pdf/inforce/current/act‐1998‐001
SA Act	*Tobacco and E‐Cigarette Products Act 1997 (SA)*. Version: 31.8.2023. Effective: 31 August 2023 [cited 7 Dec 2023]. Available from: www.legislation.sa.gov.au/lz?path=%2FC%2FA%2FTobacco%20and%20E‐Cigarette%20Products%20Act%201997
SA Act (No. 2)	*Tobacco and E‐Cigarette Products (E‐Cigarette and Other Reforms) Act 2024* (SA). Assented to: 7 November 2024 (Uncommenced) [cited 13 Nov 2024]. Available from: www.legislation.sa.gov.au/lz?path=/b/current/tobacco%20and%20e‐cigarette%20products%20(e‐cigarette%20and%20other%20reforms)%20amendment%20bill%202024
SA Reg	*Tobacco and E‐Cigarette Products Regulations 2019 (SA)*. Version: 31.8.2023. Effective 31 August 2023 [cited 7 Dec 2023]. Available from: www.legislation.sa.gov.au/lz?path=%2FC%2FR%2FTobacco%20and%20E‐Cigarette%20Products%20Regulations%202019
Tas Act	*Public Health Act 1997 (Tas)*. Effective: 24 October 2022 [cited 7 Dec 2023]. Available from: www.legislation.tas.gov.au/view/whole/html/inforce/current/act‐1997‐086
Tas Guidelines	*Guidelines for the Sale of Smoking Products* (Tas). Effective 29 November 2017 [cited 18 Nov 2024]. Available from: www.health.tas.gov.au/sites/default/files/2021‐11/Guidelines_for_sale_of_smoking_products_DoHTasmania2017.pdf
WA Act	*Tobacco Products Control Act 2006 (WA)*. Act No: 005 of 2006. Version 02‐k0‐00. Effective: 5 April 2023 [cited 7 Dec 2023]. Available from: www.legislation.wa.gov.au/legislation/statutes.nsf/main_mrtitle_983_homepage.html
WA Reg	*Tobacco Products Control Regulations 2006 (WA)*. Effective: 1 July 2023 [cited 7 Dec 2023]. Available from: www.legislation.wa.gov.au/legislation/statutes.nsf/main_mrtitle_2116_homepage.html
Vic Act	*Tobacco Act 1987 (Vic)*. No. 81 of 1987. Authorised Version No. 099. Effective: 1 September 2023 [cited 7 Dec 2023]. Available from: www.legislation.vic.gov.au/in‐force/acts/tobacco‐act‐1987/099
s	Section of Act or Regulation
div	Division of Act or Regulation
pt	Part of Act or Regulation

The Commonwealth Government has recently consolidated the provisions from several tobacco control laws into one Act (*Cth Act*). Key Commonwealth provisions related to retailing tobacco that have a focus on measures that would protect youth from tobacco marketing within physical retail environments are summarised in Table 2. Similarly, Table 3 summarises the key provisions of the Australian state and territory laws that protect youth from tobacco marketing in retail environments.

**TABLE 2:** Key commonwealth tobacco control laws related to tobacco retailing.

Regulatory provision	Description
Licensing	Not covered
Specialist tobacconists	Not covered
Sale to minors	Not covered
Purchasing on behalf of minors	Not covered
Sales by minors	Not covered
Controlled purchase operations	Not covered
Minimum pack size	A cigarette pack must contain 20 cigarettes (*Cth Act s 76; Cth Reg s 45, s 48*)
Flavoured tobacco products	From 1 July 2025 additives that have flavouring properties or that enhance flavour will be prohibited ingredients. Menthol, including synthetic menthol will be a prohibited ingredient. Devices that are capable of altering the flavour, smell or intensity of a product, including flavour capsules, will be prohibited (*Cth Act s 87–88; Cth Reg s 121, 123*). Tobacco product accessories capable of altering the flavour or smell of the tobacco product are prohibited (*Cth Act s 91*)
Packaging restrictions	Tobacco products must be sold in packaging that complies with specifications outlined in the *Cth Act* (*pt 3.3 div 1*) and *Cth Reg* (*pt 3.2–3.11*) including plain packaging with graphic health warnings, and must not produce noise or smell (*Cth Act s 83*). Prohibited terms, or any marks not permitted by regulations, must not appear on tobacco products (*Cth Act s 86*)
Toys and confectionery resembling tobacco products	A permanent ban on novelty cigarettes and toy‐like novelty cigarette lighters (*Competition and Consumer Act 2010—*Consumer Protection Notice No. 15 of 2011—Permanent ban on novelty cigarettes (F2011L00225) and Consumer Protection Notice No. 18 of 2011—Permanent ban on toy‐like novelty cigarette lighters (F2011L00228)
Display/sale of toys/confectionery/children's products in close proximity to tobacco or price board	Not covered
Retail display of tobacco products	S 20 of *Cth Act* could be interpreted as banning retail displays of tobacco products if they are considered to be a tobacco advertisement, as they are in state and territory laws, and if not permitted by a state or territory law (*Cth Act* s 31)
In‐store advertising	Banned unless allowed by State or Territory law and not visible from outside the premises (*Cth Act s 20–25, 30–31*)
Other product restrictions	Regulations can be made to specify requirements for appearance of, physical features of (e.g., cigarette dimensions), and images or text on tobacco products (*Cth Act s 86*). The content of tobacco products can be restricted and performance standards and testing requirements can be set via regulations (*Cth Act s 87, 89–90*)

**TABLE 3:** Key Australian state and territory tobacco control laws and regulations related to tobacco retailing.

Regulatory provision	State or territory							
	ACT	NSW	NT	Queensland	SA	Tasmania	WA	Victoria
Licence scheme	*Yes* (*ACT Act Pt 7*)	*Yes, but uncommenced* ^a^; retailer registration scheme still operational (*NSW Act pt 5 div 3*)	*Yes* (*NT Act pt 4*)	*Yes* (*Qld Act pt 2*)	*Yes* (*SA Act pt 2*)	*Yes* (*Tas Act pt 4 div 3*)	*Yes* (*WA Act s 16*)	*Yes, but uncommenced* ^b^
Specialist tobacconists category (minimum % sales from tobacco)	*No*	*Yes* (80%) (*NSW Act sched 1 pt 2*)	*Yes* (85%) (*NT Act s 29(5)*)	*Yes* (80%) (*Qld Act s 96(2)*)	*Yes* (80%) (*SA Reg s 7(4)*)	*Yes* (100%) (*Tas Act s 72B*)	*Yes* (80%) (*WA Act s 23*)	*Yes* (80%) (*Vic Act s 3, pt 2A*)
Sale to minors prohibited	*Yes* (*ACT Act s 14*)	*Yes* (*NSW Act s 22*)	*Yes* (*NT Act s 42*)	*Yes* (*Qld Act s 66–67, 69*)	*Yes* (*SA Act s 38A*)	*Yes* (*Tas Act s 64*)	*Yes* (*WA Act s 6*)	*Yes* (*Vic Act s 12*)
Purchasing on behalf of minors prohibited	*Yes* (*ACT Act s 15*)	*Yes* (*NSW Act s 23*)	*Yes* (*NT Act s 43*)	*No*	*No*	*No*	*Yes* (*WA Act s 7*)	*Yes* (*Vic Act s 12*)
Sale by minors prohibited	*No*	*No*	*Yes* (*NT Act s 42A*)	*Yes*; in progress^c^	*No*	*No*	*Yes* (*WA Act s 18A*)	*No*
Controlled purchase operations	*Yes* (*ACT Act pt 6A*)	*Yes* ^d^	*No*	*No*	*Yes*; in progress (*SA Act* s 39)^e^	*Yes* (*Tas Act s 67A*)^f^	*Yes* (*WA Act pt 4 div 6*)	*Yes* ^g^
Minimum pack size of 20 cigarettes	*Yes* (*ACT Act s 19*)	*Yes* (*NSW Act s 6*)	*Yes* (*NT Act s 13*)	*Yes* (*Qld Act s 84*)	*Yes* (*SA Act s 30*)	*Yes* (*Tas Act s 68(2)*)	*Yes* (*WA Act s 21*)	*Yes* (*Vic Act s 14*)
Flavours that appeal to minors prohibited (excluding menthol)	*Yes* (*ACT Act s 21‐22*)^h^	*Yes* (*NSW Act s 29*)^i^	*Yes*; licence condition bans confectionery/fruit‐flavoured/scented products (*NT Act s 29‐30*)^j^	*Yes* (*Qld Act s 164*)	*Yes* (*SA Act s 34A*)^k^	*Yes* (*Tas Act s 68A(d)‐(e)*)	*Yes* (includes mint) (*WA Act s 21B*)	*Yes* (*Vic Act pt 2 dv 5, s 15O(1), (2)(a)(i)*)^l^
Packaging restrictions	Ban orders can be made if package or packaging may be attractive to children (*ACT Act s 21–22*)	*Yes*; Must not appeal to children, for example, cartoons, holograms (*NSW Reg s 13*) Must be sold in original packaging with the required health warning and packaging must not contain prohibited words (*NSW Act div 1*)	*Yes*; pack must display prescribed health warning and packaging must not include prohibited words (*NT Act s 12; NT Reg s 16*)	*Yes*; supplier must sell tobacco products in the manufacturer or importers' package (*Qld Act ss 84(1), 85(1), 86(1)) and scheme 1 definition of ‘package’*)	*Yes* (*SA Act ss 31 and 4 definition of ‘prescribed packaging requirements’*); a person must sell or supply tobacco products that they know or ought reasonably to know does not comply with packaging requirements	*Yes* (*Tas Act ss 68(a)(ii) and 73; Tas Guideline*s *Part F(1)*); a person must not supply cigarettes to the public unless in a package. Manufactures and distributors must ensure that packages comply with any relevant guidelines	*Yes* (*WA Act* s 19; *WA Reg s 32*); a tobacco product must be in a package that is labelled in accordance with regulations	*Yes* (*Vic Act ss 15 N and 15O2(a)(ii)*; ban order can be made if tobacco product has packaging that appeals to children or young people
Toys/confectionery resembling tobacco products prohibited	*Yes* (*ACT Act s 18*)	*Yes* (*NSW Act s 21(3)*)	*Yes* (*NT Act s 46*)	*Yes* (*Qld Act s 163*)	*Yes* (*SA Act s 36*)	*Yes* (*Tas Act s 68A*)	*Yes* (*WA Act s 106*)	Ban orders can be made (*Vic Act s 15 N‐O*)
Restrictions on display/sale of toys/confectionery/children's products in proximity to tobacco or advertising/price boards	*No*	*No*	*Yes*; no priceboard within 1 m of children's products (*NT Reg s 19(6)*)	*No*	*No*	*Yes*; no tobacco sales units within 75 cm of confectionery/child products or any other product designed or marketed for children (*Tas Act* ss 71(4) and 72A(1)); specialist tobacconists cannot sell non‐tobacco related items (*Tas Act s 3*)	*Yes*; no tobacco products/smoking implements within 1 m of confectionery/child products (*WA Reg s 34*)	*Partial*; specialist tobacconists cannot sell products for children/adolescents, dairy products, bread/bakery products, or breakfast cereals^g^
Display of tobacco and related products prohibited	*Yes* (*ACT Act s 9–10, 20*)	*Yes*; limited exemptions for specialist tobacconists (*NSW Act s 9*)	*Yes* (*NT Act s 20*)	*Yes*; limited exemptions for specialist tobacconists (*Qld Act pt 4 div2*)	*Yes* (*SA Act s 40, SA Reg s 7*)	*Yes*; limited exemptions for specialist tobacconists (*Tas Act s 70, 72A, 72B*)	*Yes*; limited exemptions for specialist tobacconists (*WA Act s 22–23, WA Reg s 36–37*)	*Yes*; limited exemptions for tobacconists/airport duty‐free stores (*Vic Act s 3B, pt 2 div 2*)
Advertising prohibited, including at point‐of‐sale	*Yes* (*ACT Act* ss 4 and 23); price ticket allowed and sign informing that tobacco products for sale	*Yes* (*NT Act* s 16; *NSW Reg* ss 10 and 11); price ticket and price board allowed	*Yes* (*NT Act* ss 15 and 18; *NT Reg* s 19); price board allowed; however, it cannot be within 1 m from a display of children's products, for example, toys and confectionary	*Yes* (*Qld Act* ss 90, 93–94 and 97; *Qld Reg* ss 4 and 8); price tickets and a sign informing that tobacco products for sale allowed	*Yes* (SA Act s 40; SA Reg s 7(1)(a), (1)(g), (1)(j) and (1)(k)); price boards and price tickets allowed. Exemptions for signs informing that tobacco products for sale and tobacco products only sold in cartons.	*Yes* (*Tas Act* s 72A(3); *Tas Guidelines* cl 4 and 5); price ticket and price board allowed	*Yes* (*WA Act* ss 24 and 31; *WA Reg* ss 39–45); information signs and price tickets allowed	*Yes* (*Vic Act* s 6(3)(ca), (cab), (cb) (d), 6A; *Vic Reg* ss 7, 9, 11, 16); price tickets allowed and sign that tobacco products for sale. Exemptions for point of sale advertisement for specialist tobacconists and on‐airport duty‐free shops
Other restrictions related to protecting children								No tobacco sales at underage music/dance events (*Vic Act s 15J*)

Abbreviations: ACT, Australian Capital Territory; NSW, New South Wales, NT, Northern Territory; SA, South Australia; WA, Western Australia.

^a^ On 2 December 2024, the Parliament of NSW enacted the *Public Health (Tobacco) Amendment Act (No. 2) 2024* introducing a tobacco retailer licensing scheme [17]. Once in force, it will replace the existing retailer registration scheme.

^b^ Victoria enacted a tobacco retailer licensing scheme on 3 December 2024—*Tobacco Amendment (Tobacco Retailer and Wholesaler Licensing Scheme) Act 2024*. The act is yet to commence operation [18].

^c^ Commences 1 September 2025 for small businesses (<20 employees) and 1 September 2024 for all other businesses [19].

^d^ NSW Health Retailer Factsheet [20].

^e^ Controlled purchase operations were previously conducted by SA Health, but compliance enforcement transferred to Consumer and Business Services on 1 July 2024, which did not conduct controlled purchase operations; uncommenced provision provides for the authorisation of a minor to be a controlled purchase officer.

^f^ Department of Health (Tas) Tobacco Control Plan Progress Report 2021 [21].

^g^ Vic Department of Health and Human Services. Tobacco retailer guides [22, 23] and Annual Report 2022–2023 [24].

^h^ Tobacco (Prohibited Smoking Products) Declaration 2011 (No. 1) ACT Notifiable Instrument NI2011‐584.

^i^ NSW Government Gazette Notice [25]; NSW Government Gazette Notice [26].

^j^ NT Minister for Health [27].

^k^ SA Government Gazette Notice [28].

^l^ Vic Government Gazette Notice [29].

### 3.1 Licensing

There is no federal tobacco retailer licensing scheme (Table 2). Rather, tobacco retail licensing is the responsibility of the individual states and territories. Currently, five jurisdictions—Queensland, WA, SA, Tasmania and NT—require tobacco retailers to hold a valid licence to sell tobacco (Table 3). One state (NSW) operates a tobacco retailer notification scheme (retailers must notify the state health department of intention to sell tobacco products, but do not pay a fee) instead of a licence scheme. However, on 2 December 2024, NSW enacted the *Public Health (Tobacco) Amendment Act (No 2)* 2024, and Victoria enacted the *Tobacco Amendment (Tobacco Retailer and Wholesaler Licensing Scheme) Act* 2024 on 3 December 2024, establishing tobacco retail licensing schemes in those states (currently uncommenced). The recently enacted *Tobacco and E‐Cigarette Products (E‐Cigarette and Other Reforms) Amendment Act* 2024 (SA) amended the previous licensing provisions in SA by: (i) increasing penalties for selling tobacco without a licence (s 6); (ii) introducing a stringent character test that requires the Minister to be satisfied that the applicant is a fit and proper person to hold a licence (ss 5 and 6); and (iii) requiring the Commissioner of Police to inform the Minister if the applicant has any criminal convictions relevant to their application (s 6).

All jurisdictions with a licensing or notification scheme provide for cancellation or non‐renewal if the retailer contravenes provisions in the relevant legislation, including supplying tobacco to children. However, publicly available data on how many licences are cancelled or not renewed and the associated reasoning for the non‐granting of a licence are limited. Hence, it is difficult to ascertain how often licences have been cancelled for selling to children. For example, the Tasmanian Government reports that ‘the number of licensed tobacco retailers declined from 729 in 2017 to 635 in 2021’ [21], but does not provide any breakdown between licensees who voluntarily surrendered or failed to renew their licence and those who had their licence suspended or cancelled. Similarly, we found no recent reported decisions from any of the administrative tribunals overseeing decisions not to renew a licence so it is unclear how regularly licence provisions are cancelled as a result of the commission of an offence of supplying to minors. However, an absence of reports does not confirm that no licence cancellation or non‐renewal decisions have been made.

The licensing schemes also permit the imposing of conditions on licensees which operate to regulate the sale of tobacco products in retail outlets. However, there are points of difference concerning the nature and scope of these provisions between jurisdictions. For instance, in SA, the Minister is authorised to prescribe conditions on a retail tobacco licence restricting where tobacco products can be sold on the premises (*SA Act* s 9(2)(b)). Similarly, in WA retailers must ensure that tobacco products are only sold in one place on the licensed premises (*WA Act* s 20(1)). In the NT, sale of fruit and confectionary‐flavoured tobacco products is not permitted via a condition imposed on all licences (*NT Act* s 30; Scholz D, NT Health, 2023, personal communication). In Queensland, the licensing provisions allow the Chief Executive to impose specific conditions on tobacco licences instead of suspending or cancelling the licence (*Qld Act* ss 33). Relevantly, in WA, it is a general condition that retail licensees instruct employees and agents not to sell a tobacco product to a child or a person they believe is not over 18 years of age unless a valid proof of age document is presented (*WA Reg* s 20(1)).

### 3.2 Specialist tobacconists

Apart from the Australian Capital Territory and Queensland, most jurisdictions with a licensing scheme currently have a licence category for specialist tobacconists. Notably, the *Qld Act* defines specialist tobacconists, but does not have a separate licence category for them. To be considered a specialist tobacconist, over 80% of general turnover in NSW, Queensland, SA, Victoria and WA (85% in the NT and 100% in Tasmania) must be derived from tobacco and the tobacco business must be conducted separately from other businesses. The specialist tobacconist category has generally been part of transitional arrangements when new restrictions on tobacco retail displays were introduced (see, e.g., *WA Act* s 23). Victoria allowed retailers to apply for certification as a specialist tobacconist until 1 April 2014 to permit these existing retailers to continue displaying some tobacco products and packs after new display bans commenced on 1 January 2011 [23]. Some states exempt specialist tobacconists from some marketing restrictions. For example, WA allows specialist tobacconists to display cigars (*WA Reg* s 23(2)), Queensland allows them to include tobacco‐related words in their business names (*Qld Act* s 95(1)), and SA allows them to display a larger tobacco price board than other retailers (*SA Reg* s 7(1)(j)). However, the broad suite of tobacco advertising, promotion, and sponsorship restrictions, such as in‐store advertising bans continue to apply, and price‐related terms cannot be combined with tobacco terms for business names and signage (e.g., Queensland prohibits names, such as Discount Cigs and Tobacco; *Qld Act* s 95(2)). Although restrictions are still in place for specialist tobacconists, many of these exceptions expose children to tobacco products and there are no provisions to restrict entry of children to the premises.

### 3.3 Sale to minors

All states and territories ban the sale of tobacco products to minors under the age of 18 (Table 3). The *SA Act (No. 2)* seeks to ‘take steps to eliminate the uptake of smoking … by young people’ by strengthening the existing legislative framework. For instance, a person convicted of selling or supplying a tobacco product to a child will be liable for a fine of up to $1,000,000 for a corporation, and $500,000 for an individual for a first offence (*SA Act (No. 2)* s 18(3)), which is a substantial increase from the previous version of the Act (*SA Act* s 38A). In Queensland, the *Tobacco and Other Smoking Products (Vaping) and Other Legislation Amendment Act 2024* commenced operation on 19 September 2024. The new legislation amends the existing law by extending criminal liability to executive officers of a corporation if they, or an employee, supplies a smoking product to a child (s 11, s 12 and s 31).

### 3.4 Purchasing on behalf of minors

Purchasing on behalf of minors is prohibited in five jurisdictions, and offenders can face significant penalties for contravening the prohibition (see, *Qld Act* s 82(1); *NT Act* ss 7 and 115).

### 3.5 Sales by minors

Minors are permitted to sell cigarettes in all jurisdictions except WA, NT and Queensland (currently being phased in). In SA, it is illegal for a person under 16 years of age to sell or supply cigarettes (S*A Act* s 39D).

### 3.6 Controlled purchase operations

All jurisdictions except Queensland and the NT report using routine covert controlled/test purchase operations to monitor compliance with age restrictions on tobacco sales. In SA, the controlled purchase operation provisions commenced on 13 December 2024 (*SA Act (No. 2)* s 39). Victoria's enforcement activities, including covert monitoring, are publicly reported annually, including outcomes and when targets are not met [24].

### 3.7 Minimum pack size

The sale of single cigarette sticks is banned in all jurisdictions, and a minimum pack size of 20 cigarettes is mandated (Table 3). These measures reduce the affordability of tobacco products for children because children typically can only access small amounts of money, which makes paying a larger upfront purchase price more difficult. From 1 July 2025, the new federal tobacco control regulatory reforms will standardise the size of a cigarette pack to 20 cigarettes nationally (Table 2).

### 3.8 Flavour prohibitions

The sale or supply of fruit‐ or confectionary‐flavoured traditional tobacco products is prohibited in all states and territories (Table 3; e.g., *WA Act* s 21B(a)). One jurisdiction, WA, also prohibits mint‐flavoured tobacco products (*WA Act* s 21B(b)). Victoria's regime allows the Health Minister to ban the sale of tobacco products (and packaging) that appeal to minors. In 2011, the Victorian Minister for Health used this power to ban several types of tobacco products (cigarette papers, cigar wraps, cigarillo wraps and cigarillos) that have a fruity, sweet or confectionery‐like flavour or scent [29]. From 1 July 2025, flavoured tobacco products are also prohibited by Commonwealth legislation (Table 2). For example, additives that have flavouring properties or that enhance flavour are prohibited ingredients (*Cth Reg* s 121). This will include menthol and synthetic menthol (*Cth Reg* s 121, item 9). Flavour capsules or other devices that can alter the flavour, smell or intensity of a product, are also prohibited (*Cth Reg* s 123).

### 3.9 Packaging restrictions

The *Cth Act* sets a standard for packaging across all jurisdictions in Australia that includes plain packaging with large graphic health warning labels (*Cth Act* Ch 3, Pt 3.3, Div 1; *Cth Reg* Ch 3). The sale of tobacco products in non‐conforming packaging is also banned by some state and territory laws. For example, in NSW and the NT, it is an offence to sell a tobacco product that does not conform with the *Cth Act* regarding prescribed health warnings (*NT Act* s 12(1); *NT Reg* s 16; *NSW Act* ss 5 and 7(1)). Similarly, in Tasmania, WA and SA it is an offence to sell tobacco products in packaging that does not comply with *Cth Act* (Table 3; e.g., *Tas Act* s 73; *Tas Guidelines* Part F; *WA Act* s 19; *WA Reg* s 32). Although there is some overlap between the *Cth Act* and the individual state and territory laws concerning tobacco product packaging, it is important to bear in mind that the *Cth Act* sets the standard for packaging requirements that applies across all Australian jurisdictions, and the states and territories retain the power under their legislative framework to prosecute offenders for selling non‐compliant tobacco products.

### 3.10 Sale of toys/confectionery/children's products and other products resembling tobacco products

The sale of ‘toys or products that resemble tobacco products’ is banned in all jurisdictions other than Victoria (Table 3; e.g., see *WA Act* s 106; *Tas Act* s 68(a)). NT's provision clarifies the prohibition through the consideration of intent, prohibiting the sale of any product that ‘resembles a tobacco product’ or ‘has the effect of encouraging children to smoke (whether it is intended to have that effect or not)’ (Table 3; *NT Act* s 46). There are permanent bans implemented under the *Competition and Consumer Act 2010 (Cth)* on novelty cigarette lighters due to their appeal to children and the related fire risk, and on novelty cigarettes due to the health risk posed by an ingredient (hydrated magnesium silicate). Sale or display of toys, confectionery or other products that resemble tobacco products may also be banned under the new *Cth Act* (s 20), if sale/display of these products is deemed to constitute publication of a tobacco advertisement under the broad definitions within the Act, and if they are not expressly permitted under state and territory laws (Table 2).

### 3.11 Display/sale of toys/confectionery/children's products in close proximity to tobacco or price boards

WA and Tasmania have restricted the location of the display of specific non‐tobacco products, including food and toys, close to tobacco in general tobacco retailer outlets (Table 3; e.g., *WA Act* s 34(5)(a)). In the NT, tobacco price boards must not be positioned within 1 metre of children's products, including toys and confectionery (*NT Reg* s 19). Under the *Tas Act*, it is an offence to display a tobacco product within 75 cm of confectionary; however, an exemption can be granted if the distance cannot be practically maintained (s 70(3)). Furthermore, specialist tobacconists in Tasmania can only sell tobacco and related products; hence, no children's products or food can be sold by them (*Tas Act* s 72B). Under the current Victorian framework, certified specialist retailers are prohibited from selling ‘products or services for children or adolescents’ including confectionery and toys and ‘dairy products, bread and bakery products, breakfast cereals or food or beverages other than low‐risk shelf stable foods and beverages’ (Table 3).

### 3.12 Tobacco product displays

All jurisdictions ban tobacco product displays (Table 3; e.g. *ACT Act s 9–10, 20*).

### 3.13 In‐store advertising

All jurisdictions ban in‐store tobacco advertising with some exceptions permitted (Table 3; e.g. *Qld Act* s 90(2), (3)). For instance, price tickets and signs stating that tobacco products are sold by retailers are allowed providing they adhere to strict standards (Table 3; e.g., *SA Act* s 40; *SA Reg* s 6; *Qld Act* s 94; *Qld Reg* s 4; *WA Act* ss 24 and 32(1)(d); *WA Reg* ss 40 and 41). In SA, the advertisement of a tobacco product on a price board is permitted if it complies with the design and information specifications set out in the *SA Regs* (s 7(1)(j)) and the price board does not exceed 1 m^2^ for a specialist tobacconist and 0.5 m^2^ for other licensed retailers (s 7(1)(j)(ii)(A), (B)). In Queensland, advertising the retail price of tobacco products is permitted, however, the price ticket cannot indicate that the price is discounted (*Qld Act* s 94). In the NT, a single price board is permitted at the point‐of‐sale, providing that it complies with the *NT Regs* regarding design and information specifications (*NT Reg* s 19).

Certified specialist retailers in Victoria are restricted from having more than four metres squared of ‘accessory products.’ Accessory products are also not permitted to be advertised in a way that is designed to appeal to children.

### 3.14 Other restrictions

Victoria specifically prohibits tobacco product sales at underage dance and music events irrespective of the purchaser's age (*Vic Act* s 15J).

## 4 DISCUSSION

Our study reviewed Australian laws that address tobacco product retailing with a focus on protecting children from tobacco marketing. The most comprehensive and nationally consistent laws are a ban on tobacco sales to minors, a minimum pack size of 20 cigarettes, plain packaging requirements, and a ban on fruit or confectionery flavoured tobacco products. For some, national consistency is achieved through federal legislation (e.g., plain packaging, tobacco flavouring bans and standard pack size), but others are achieved by all states and territories adopting similar provisions (minimum purchase age and minimum pack size). Extensive bans on in‐store advertising and tobacco product displays are also included in all sub‐national laws with minor exemptions for specialist tobacconists in some jurisdictions and allowance for price and business signage. There is overlap in some tobacco control laws at state/territory and federal level (e.g., minimum pack size, flavoured tobacco product bans and advertising bans). In a federal system of government like Australia, an overlap between state/territory and federal legislation is not uncommon. Where an overlap of law results in an inconsistency between state/territory and federal legislation, the Australian Constitution resolves this inconsistency in favour of the federal law, invalidating the respective state or territory law to the extent of the inconsistency (*Australian Constitution 1901*s 109). An illustration of this is evident in WA. The *WA Act* permits the sale of menthol flavoured cigarettes (s 21B(b)), but the *Cth Act* will prohibit them from 1 July 2025 (*Cth Reg* s 121). As this is a clear inconsistency, the federal legislation prevails, and menthol flavoured cigarettes will be prohibited in WA. However, the *Cth Act* specifically acknowledges that it ‘does not exclude or limit the operation of state or territory law that is capable of operating concurrently (*Cth Act* s 7(1)),’ thus providing necessary clarification on matters like enforcement for contraventions against the respective legislation. This allows the states and territories to prosecute offenders for violations under their local legislation. However, if charges are brought against an offender under the federal legislation, then this is the responsibility of the federal government. To avoid duplication of liability, the *Cth Act* clarifies that an offender cannot be charged again under the federal legislation if they have already been punished for a similar offence under the state or territory law (s 7(4)).

We found significant opportunities to strengthen current laws. For example, two jurisdictions (Victoria and NSW) only recently introduced tobacco retail licensing schemes (both yet to commence operation) as part of a response to a growing illicit tobacco market [17, 18]. On 29 August 2024, the Public Accounts and Estimates Committee tabled its inquiry on Vaping and Tobacco Controls in the Parliament of Victoria [30]. The Public Accounts and Estimates Committee made 27 recommendations in total and ‘at its centrepiece [recommended] the establishment of a Victorian nicotine licensing scheme and active regulatory authority’ [30]. The committee also recommended restricting the number of licences granted, and applying ‘density limits for each local government area, and prohibition of licenses within 150 m of a school’ [30]. However, no density or proximity limits on tobacco retail licences were included in the Victorian licensing scheme.

Research has shown a state government tobacco retailer list in NSW [31] and a local government's tobacco retailer list in Victoria are inaccurate, highlighting the challenges in monitoring tobacco retailing laws without a retailer licensing scheme [32]. Positive tobacco retail licensing schemes, that is, the requirement for retailers to be licensed before selling tobacco, are considered best practice [33] because they enable education of retailers as part of the application process; create a list of current tobacco retailers; send a message that selling tobacco (a hazardous product) is a privilege not an unconditional right; enable a range of cost‐effective enforcement options (e.g., licence conditions, licence suspension and licence cancellation); can include a character test for licence holders; generate funds from licence fees for monitoring and enforcement; and can discourage retailers from selling tobacco [33, 34]. Furthermore, details on the annual number of new licences issued, voluntarily surrendered, not renewed or suspended/cancelled (and for what reasons), should be publicly reported by area in all jurisdictions. This will provide transparency in the operation of tobacco retail licensing schemes and provide data on the number of retailers, changes in tobacco retailing (e.g., anecdotal reports suggest a rapid rise in new tobacconists has corresponded with the recent rapid increase in illicit tobacco use) and whether retailing laws are being enforced [33].

Brown et al. identified four key retailing strategies that the tobacco industry uses to target and increase the youth market including ‘the display of cigarettes near snacks, sweets and sugary drinks, placement of cigarette advertisements near the eye‐level of children, advertisements and display of flavoured cigarettes and sale of single sticks of cigarettes’ [35]. Australian laws cover these four aspects, and our review provides examples of how these marketing practices are restricted in Australia that may be of interest to other jurisdictions internationally that have not implemented similar restrictions. However, we also identified numerous gaps in Australia's tobacco retailing regulation that leave children exposed to tobacco marketing and undermine tobacco denormalisation strategies. Examples are that some jurisdictions allow minors to sell tobacco, and none restrict the location of tobacco retailers or restrict the use of child‐friendly imagery or product displays (e.g., toys and confectionery) at the entrance or windows of tobacconists. Furthermore, in the absence of a consistent regulatory response across jurisdictions, Australia lacks a coherent national approach despite federal policy statements that support ‘a consistent whole‐of‐government approach … to tobacco control in Australia,’ such as ‘a consistent licensing scheme in place covering all aspects of the tobacco supply chain in Australia’ [36]. However, two states still lack an operational licensing scheme, although both are in the process of implementing one [17, 18]. As previously discussed, there are differences between how each licensing scheme (both existing and in progress) operate between jurisdictions. While states and territories are responsible for implementing tobacco retail licensing, achieving national consistency in licensing, a goal of the National Tobacco Strategy [36], could be achieved through a licensing standard implemented at the federal level, that is then adopted through state and territory laws. This process could be modelled on the standardised approach to categorisation and regulation of medicines and poisons that is achieved via the Commonwealth Poisons Standard [37], which is enacted through state and territory laws. Exploring mechanisms to achieve a consistent licensing scheme is a priority action item in the National Tobacco Strategy that was endorsed by all Australian Health Ministers [36].

As noted, there are no regulatory provisions in any Australian state/territory that restrict the location of tobacco retailing, including that of specialist tobacconists, who are exempt from some tobacco advertising, promotion, and sponsorship restrictions. Tobacco retailing is prevalent in most retailing precincts in Australia, including shopping centres and malls, making tobacco retailers widely visible, accessible and attractive to children and minors, often explicitly through their signage, window displays and location close to youth‐focused retailers [38]. Internationally, some jurisdictions have banned tobacco sales within set distances from schools or other educational facilities [39, 40]. The only Australian example of a current restriction on the location of tobacco retailing we found was a ban on tobacco sales at underage music/dance events in Victoria. While some Australian educational facilities have campus‐wide sales bans (e.g., The University of Queensland [41], University of Canberra [42]), these are implemented via these institutions' policies, such as via lease agreements for on‐campus retailers, not legislation. Australia's National Tobacco Strategy includes the priority action item of exploring ‘options to further regulate where tobacco products are retailed, including regulatory approaches to control or restrict the number, type and location of tobacco outlets’ [36]. We recommend that protection of children be considered as part of this potential policy approach, such as considering proximity of outlets near youth‐focused locations, particularly schools.

Anecdotal evidence suggests that some Australian tobacconists are undermining tobacco control measures to protect children from tobacco retailing by selling confectionery, toys and other products designed to appeal to minors and using youth‐friendly imagery in signage [38]. Examples include a tobacconist featuring images of the PG rated movie ‘The Mask’ in its signage and selling ‘American candy’ [43], a tobacconist store painted in ‘SpongeBob’ cartoon images [44], a tobacconist featuring a large ‘Pikachu’ Pokémon character at its entrance [38] and tobacconists with window toy displays at child height [38]. Emerging research suggests that adolescent exposure to tobacco‐related products is associated with increased long‐term tobacco use, even when exposure is not directly to cigarette products [45]. Prohibiting tobacconists from attracting children to their stores through toy or confectionery window and entrance displays and preventing children from entering tobacconists thus present two opportunities to reduce children's exposure to tobacco marketing. Further, stores that predominantly sell children's products, such as toy stores, have been found selling tobacco in Australia [46], which is not prevented by current tobacco retailing regulatory frameworks.

Proximity of tobacco and products that appeal to children may lead youth to conflate the acceptability of these products with tobacco and contribute to their misperceptions of the health risks and normality of tobacco products [47]. Prohibiting mixed‐product retailing that includes tobacco and child‐friendly products would counteract this. At a minimum therefore, we recommend that all jurisdictions strengthen current laws throughout Australia to separate tobacco product sales from sale of children's products. Examples of possible regulatory approaches include a minimum distance between toy or confectionery displays and tobacco sales units/counters or price boards, similar to in‐store proximity provisions currently in place in the NT, WA and Tasmania. Retailers who make the majority of their turnover from retailing tobacco products could be prohibited from selling products intended for children and adolescents and confectionery. Similarly, retailers who primarily sell products intended for children and adolescents could be prohibited from retailing tobacco.

These regulatory options represent only incremental steps that will still leave many young people exposed to tobacco retailing. To more effectively protect youth from exposure to tobacco retailing, Australian governments should work towards completely separating the supply of tobacco products from the retailing of ordinary commodities and phasing tobacco out of all general/mixed business retailing environments, including supermarkets, as has been done in the Netherlands [48]. While proximity limits on tobacco retailers and prohibiting co‐retailing of tobacco and youth‐oriented products may seem substantial restrictions compared to the current level of regulation on smoked tobacco products in Australia, they are far less than those imposed on vaping products, non‐smoked tobacco, and other non‐therapeutic nicotine products. All vaping products are now regulated in Australia as therapeutic goods, regardless of their nicotine content. Hence, supply, advertising and promotion of vaping products are restricted through state/territory medicines and poisons legislation that follow a federal standard (the Poisons Standard) [49] with some minor variations, and federally by the *Therapeutic Goods Act* 1989 (Cth) [50]. Substantial regulatory reforms were implemented federally in 2024 to protect ‘young Australians’ from vaping products [51]. These reforms banned the importation of disposable vaping products and restricted the importation of all other vaping products (including nicotine‐free products) to importers with a permit from the Office of Drug Control [51]. Vaping product supply outside of the therapeutic pathway (medical practitioners, nurse practitioners and pharmacists) was banned nationally from 1 July 2024 [51]. Oral nicotine products made from extracted or synthesised nicotine are also regulated as therapeutic goods and cannot be sold by general retailers unless they have been approved by the medicines regulator, the Therapeutic Goods Administration [52]. Commercial importation of chewing tobacco and oral snuff was banned federally in 1991 [53], some state and territory tobacco control laws also ban their sale or supply (*Qld Act* s 162, *SA Act* s 25, *Vic Act* s 15), and the new *Cth Act* also bans the commercial importation, supply and possession of chewing tobacco and oral snuff (s 126).

Limiting tobacco sales to stores that restrict entry to adults only could also be explored as a regulatory option that would prevent children's repeated exposure to tobacco purchasing behaviours, potentially reducing the likelihood that they will initiate smoking in the future [10, 15, 39]. Unlike regimes for other age‐based restricted products, such as alcohol [54], there are no restrictions in any Australian jurisdictions for minors entering or remaining on these premises, despite the prohibition on minors purchasing tobacco products in all jurisdictions. Internationally, some local government jurisdictions have gone further than imposing age‐based entry restrictions on tobacco retailers, and have either banned the sale of tobacco products entirely within their area, or commenced a generational phase out of tobacco sales by prohibiting tobacco product sales to everyone born after a set year [48]. If passed into law, the *Tobacco and Vapes Bill* (UK), introduced to parliament in November 2024, will ban selling tobacco products to anyone ‘born on or after 1 January 2009’ in the United Kingdom [55]. In SA, an independent member of parliament, the Hon Frank Pangallo, has introduced the *Tobacco and E‐Cigarette Products (Miscellaneous) Amendment Bill 2024 (SA)*, which also proposes a ban on selling or supplying nicotine and tobacco products to anyone born on or after 1 January 2009 (s 6) [56]. Pangallo's Bill passed the Legislative Council on 25 September 2024 and is awaiting debate in the House of Assembly. An attempt to introduce a similar law in Tasmania was unsuccessful [48]. In addition to measures, such as a generational phaseout, alternative tobacco supply models, such as pharmaceutical‐like regulation or non‐profit models, should be explored to reduce the commercial incentive to maintain tobacco sales and to recruit new generations to tobacco use [57, 58]. Research on public support for more substantial regulatory reforms for tobacco retailing and also on the potential economic and practical impacts on the government and the retailing sectors and legal barriers to implementation is needed.

### 4.1 Strengths and limitations

This study provided the most comprehensive up‐to‐date review of current laws across Australia that are intended to protect children from exposure to tobacco product marketing in retail environments in a country that is considered a leader in tobacco advertising, promotion, and sponsorship regulation. Legislation is regularly amended and hence, these results represent the current situation at one point in time. However, we have also reviewed and referenced regulatory reforms that are currently in progress. We focused on tobacco control laws, so other laws that could restrict where tobacco retailers operate, such as local government planning laws, were outside the scope of this study. Given the substantial differences in bricks‐and‐mortar stores and online retailing environments, we have focused only on one of these channels (bricks‐and‐mortar) in this study. Future research should review the Australian laws that cover online retail environments.

## 5 CONCLUSION

As restrictions on traditional tobacco advertising have increased, the tobacco industry has innovated and increased its reliance on covert marketing in retail environments. Despite rhetoric from policymakers that protecting children from exposure to tobacco marketing is a priority, there are still significant opportunities for youth to be targeted by tobacco retailers and attracted to smoking through tobacco retailer practices that remain under‐regulated. Closing regulatory gaps to prevent tobacconists from selling products that are specifically designed for children, including toys, confectionery and other youth‐oriented products and services, is an example of action that would translate this rhetoric into practical action that protects children from exposure to tobacco products and would contribute to denormalisation of commercial tobacco products. Restricting entry by children to tobacconists and ending tobacco retailing by general mixed business retailers are also important measures that should be considered as part of Australian government policy goals to reduce the availability of tobacco products and to protect young people from tobacco. Implementing nationally consistent tobacco retailer licensing would help Australia meet its international obligations under the FCTC, add to ongoing efforts to denormalise smoking, and more consistently protect children from tobacco product marketing throughout the country.

## AUTHOR CONTRIBUTIONS

TR conducted the initial review of the state and territory laws and wrote the first draft. KM reviewed the federal laws and revised the manuscript. CG and MO double screened all the laws, expanded and updated the review and substantially revised the manuscript. BR provided critical revisions.

## CONFLICT OF INTEREST STATEMENT

The authors have no competing interests.

## Data Availability

All data are publicly available via the URL links provided in Table 1.
